# A Novel Gradient Vector Flow Snake Model Based on Convex Function for Infrared Image Segmentation

**DOI:** 10.3390/s16101756

**Published:** 2016-10-21

**Authors:** Rui Zhang, Shiping Zhu, Qin Zhou

**Affiliations:** Department of Measurement Control and Information Technology, School of Instrumentation Science and Optoelectronics Engineering, Beihang University, Beijing 100191, China; 13171093@buaa.edu.cn (R.Z.); qin_zhou_buaa@163.com (Q.Z.)

**Keywords:** active contour model, gradient vector flow, infrared image segmentation, external force field

## Abstract

Infrared image segmentation is a challenging topic because infrared images are characterized by high noise, low contrast, and weak edges. Active contour models, especially gradient vector flow, have several advantages in terms of infrared image segmentation. However, the GVF (Gradient Vector Flow) model also has some drawbacks including a dilemma between noise smoothing and weak edge protection, which decrease the effect of infrared image segmentation significantly. In order to solve this problem, we propose a novel generalized gradient vector flow snakes model combining GGVF (Generic Gradient Vector Flow) and NBGVF (Normally Biased Gradient Vector Flow) models. We also adopt a new type of coefficients setting in the form of convex function to improve the ability of protecting weak edges while smoothing noises. Experimental results and comparisons against other methods indicate that our proposed snakes model owns better ability in terms of infrared image segmentation than other snakes models.

## 1. Introduction

Infrared radiation is an invisible type of electromagnetic wave, whose wavelength ranges between the radio wave and the visible light. Any object in nature whose temperature is over absolute zero (−273 °C) is able to radiate the infrared ray. Compared with the visible light, the infrared wave has its unique characteristics. For example, compared with the visible light, its light quantum energy is much lower, the heat effect is stronger, it is more likely to be absorbed by a substance, and is less sensitive to the human eye.

The visible light between 0.4~0.75 μm can be sensed by human eyes. The light outside this range cannot be sensed without the aid of detectors. After in-depth studies, the interaction between the medium and the radiation source has been discovered and the infrared radiation laws have been summarized, which greatly stimulate the development of the infrared technology. The advent and development of the infrared thermal imaging system indirectly broadens the visual sensing scope of the eyes. The most commonly used detector is the infrared thermal detector. It can measure the infrared thermal radiation quantity in a non-contact manner, convert it into clearly visible images and display them on a screen.

The infrared technology was first applied for military purposes. In recent years, the technology has been widely used in transportation, medicine and other scientific areas. The on-board infrared scanning imaging technology can be used to monitor the location and affected regions of a forest fire, control a fire disaster and minimize losses. In medicine, the infrared technology can be used to detect inflamed organs and diagnose early symptoms of cancers. In the electronic equipment manufacturing industry, the quality and reliability of the electrical circuits and devices can be evaluated using the infrared technology.

Compared with the visible light images, the infrared images have the following features: (1) most objects in the infrared images have weak edges; (2) most of the infrared images have a high degree of heterogeneity; (3) the contrast of the infrared images is low; (4) there are many types and large quantity of noises in the infrared images; and (5) the resolution of the infrared images is low.

Considering the features above, the traditional methods are ineffective in segmenting the infrared images. The active contour model has the following considerable advantages in terms of infrared image segmentation: (a) The object’s edges obtained by the model is smooth, and the model is very robust to edge clearance in the image; (b) The segmentation results represent the object’s edges with closed curves, thereby dispensing with the need to connect edges of the segmentation results. The closed contour is more conducive to object analysis and recognition; (c) The partial differential equation can be used to compute the relatively mature results via theoretical and numerical analysis. The model can also directly process the features of the to-be-segmented images (e.g., curvature and gradient). Hence, the model is very robust and capable of yielding better segmentation results.

Currently, the active contour model has been widely used for segmentation of medical images [1,2,3,4,5]. This type of model has developed rapidly and its variants spring up in recent years, such as CN-GGVF [6], ADF [7] and DWP [8]. Due to the unique characteristics of the infrared images (e.g., low contrast, serious noise and non-uniform distribution), the study on the use of active contour model for segmentation of infrared images is in the infancy. Some attempts have been made to segment infrared images using active contour model [9,10,11,12,13]. Furthermore, the active contour model can be used for object tracking [14,15] and edge reconstruction [16]. Generally, there is a much work to be done on the segmentation of infrared images using active contour model. Existing study shows that the active contour provides a very promising approach for the segmentation of infrared images.

The active contour model can be classified into the edge-based models, which includes parametric models [17,18,19,20,21,22] and the geometric (or geodesic) models [14,23,24,25,26,27], and the region-based models [11,28,29]. This paper focuses on the parametric models and proposes a novel model to segment infrared images more accurately. The proposed model has advantages in terms of weak edge protection and noise smoothing. Experiments are carried out to segment the real-world infrared images using the proposed model and other traditional active contour algorithms for the purpose of evaluating accuracy and other aspects of their performance. We come to a conclusion about this paper finally.

## 2. Research Background

### 2.1. Traditional Snakes Model

In 1987, Kass and co-workers proposed an active contour model, which is also known as the Snakes model [17]. The traditional version of the active contour model is a continuous closed curve and represented with parameter curve c(q)=[x(q),y(q)],q∈[0,1]. The energy functional is minimized by moving the curve in the image, as shown in Equation (1).

(1) $$E\left(c\left(q\right)\right)=\frac{1}{2}\int_{0}^{1}\alpha {\left|c^{\prime }\left(q\right)\right|}^{2}+\beta {\left|c^{\prime \prime }\left(q\right)\right|}^{2}dq+\;\int_{0}^{1}E_{ext}\left(c\left(q\right)\right)dq$$

where α and β are the weighting coefficients that adjust the flexibility and rigidity of the curve in the active contour. The first integral term in the equation is the internal energy that ensures smoothness and continuity of the curve. The second integral term is the exterior energy, which contains the information of the image where the contour curve is located. It is a man-made constraint specifically introduced to guarantee the curve evolves towards the object contour more accurately and quickly.

In the traditional Snakes model, the external energy is usually defined as the local features of the image where the controlling point or the connecting line is located. The gradient is usually used as the feature, as shown in Equation (2).

(2) $$E_{ext}\left(x,y\right)=-{\left|\nabla I\left(x,y\right)\right|}^{2}$$

The limitations of the traditional Snakes model are as follows:

1. It is very sensitive to the location of the initial contour. During the practical segmentation process, the initial location of the contour must be manually put near the image edge of interest, resulting in poor interactivity.
2. It is prone to converge towards the false edge near the object, and is not robust to the noise.
3. Its convergence performance is poor for the object contour with sunken regions.

### 2.2. The GVF Snakes Model

The catching range of the traditional Snakes model is limited, and the external energy only exists in the regions near the object contour. Xu and Prince proposed a new external force model for the active contour, i.e., the gradient vector flow (GVF) Snakes model [18]. GVF refers to the vector field obtained by propagating the gradient vector of the edge graph for the given image. It is represented with a function and can be determined using the following dynamic evolution equation.

(3) $$V_{t}\left(x,y,t\right)=\mu \nabla^{2}V\left(x,y,t\right)-{\left|\nabla f\right|}^{2}\left[V\left(x,y,t\right)-\nabla f\right]$$

where μ is the parameter that can control the degree of smoothness of the external force field in GVF, and the value set to it increases with the noise intensity in the image. f is the edge graph of the input image. $\nabla^{2}$ is the Laplace operator. The largest advantage of GVF Snakes over the traditional Snakes model is its ability to broaden the catching range of the initial contour and to catch the high-curvature region in the object contour.

### 2.3. An Improved GVF Snakes Model

Although the GVF Snakes model has many advantages, it also has many limitations. Many variants have been presented to address the limitations.

#### 2.3.1. GGVF Snakes Model

In 1998, Xu and co-workers introduced two weighting coefficients that can change in the image domain to the iteration equation of the GVF external force field. In this way, they obtained a new external force called the generic gradient vector flow (GGVF) external force field [19]. The evolution equation of this external force field is:

(4) $$V_{t-\mathrm{ggvf}}\left(x,y,t\right)=g(\left|\nabla f\right|)\nabla^{2}V\left(x,y,t\right)-h\left(\left|\nabla f\right|\right)\left[V\left(x,y,t\right)-\nabla f\right]$$

(5) $$g\left(\left|\nabla f\right|\right)=e^{-\left|\nabla f\right|/K}$$

(6) $$h\left(\left|\nabla f\right|\right)=1-e^{-\left|\nabla f\right|/K}$$

where the parameter K determines the weight of the smooth term and the data term. As in the GVF Snakes model, the choice of K relates to the image noise. The larger the noise, the larger the value of K should be.

This model provides an approach to the problem that GVF Snakes can hardly converge towards the long narrow sunken regions and is not very robust to the noise.

Afterwards, Qin proposed an improved model of the GGVF external force field in 2013, i.e., the component-based normalized GGVF model (CNGGVF) external force field [6]. CNGGVF addresses the problem of GGVF Snakes that it can hardly converge towards LTI (Long and Thin Indentation), which is an even number of pixels in width.

#### 2.3.2. NGVF Snakes and NBGVF Snakes

The Laplace operator can be decomposed along the tangent and normal directions. Hence, the evolution equation of the GVF external force field can be rewritten as:

(7) $$V_{t}\left(x,y,t\right)=\mu \left(\alpha V_{TT}\left(x,y,t\right)+\beta V_{NN}\left(x,y,t\right)\right)-{\left|\nabla f\right|}^{2}\left[V\left(x,y,t\right)-\nabla f\right]$$

where $V_{TT}$ and $V_{NN}$ denote the second-order derivative along the tangent and normal directions of $V_{t}$. The parameters α and β determine the degree of image diffusion along the tangent and normal directions.

Normally, the interpolation method yields the best results. Based on this, Ning et al. proposed a normal gradient vector flow (NGVF) external force field [20]. From Equation (7), we can know that:

(8) α=0

(9) β=1

After their investigations, You et al. discovered that diffusion along the tangent direction of the image edge can protect the image edge, and the diffusion along the normal direction can smooth the noise. The NGVF external force field abandons the tangent diffusion, making it difficult for the NGVF Snakes model to protect the weak edge of images. In this context, Wang et al. proposed a normally biased gradient vector flow (NBGVF) external force field [21]. NBGVF completely retains the tangent diffusion and is capable of adapting normal diffusion to image structure.

To sum up, in the NBGVF Snakes model, the parameters and definitions in Equation (7) can be defined as:

(10) α=1

(11) $$\beta =g\left(\left|\nabla f\right|\right)=e^{-{\left|\nabla f\right|}^{2}/K^{2}}$$

This improved version of the model has higher diffusion efficiency and is able to effectively protect the weak edges. The weaker the edge to be protected, the smaller the value of K.

## 3. Algorithm Improvement

### 3.1. Improved Version of the GVF Model

As discussed in the section above, the GGVF Snakes model enlarges the convergence range of the active contour, improves the LTI convergence performance and is more robust to the noise. Based on NGVF, which has higher diffusion efficiency, NBGVF provides a solution to the weak edge protection problem. Hence, this paper relies on the GVF external force model, and combines GGVF and NBGVF to propose a novel external force model.

The improved version of the external force is defined as a vector field, and it can be obtained by using the following energy functional:

(12) $$E\left(V\right)=\iint g\left(x,y\right)\left(gs\left(x,y\right)V_{NN}+hs\left(x,y\right)V_{TT}\right)dxdy+h\left(x,y\right)\left(V-\nabla f\right)dxdy$$

(13) $$g\left(\left|\nabla f\right|\right)=e^{-\left|\nabla f\right|/K}$$

(14) $$h\left(\left|\nabla f\right|\right)=1-e^{-\left|\nabla f\right|/K}$$

(15) $$hs\left(f\right)=\{\begin{array}{c} 1\;\;\;\;\;\;\;\left(\left|e\right|\geq \tau \right)\\ -\frac{f^{3}}{8\tau^{3}}+\frac{5f}{8\tau }+\frac{1}{2}\\ 0\;\;\;\;\;\;\;\;\left(\left|e\right|=0\right) \end{array}\left(0<\left|e\right|<\tau \right)$$

(16) gs(f)=1− hs(f)

where $V_{NN}$ and $V_{TT}$ denote the second-order derivative along the normal and tangent directions. g(|∇f|) and h(|∇f|) denote the coefficients of the smooth and data terms in Equation (12). As defined in GGVF, the value of K increases with the noise intensity in the image, but this may lead to the weak edge being over-smoothed. Unlike the coefficients of the normal and tangent diffusion operators in NBGVF, both of the coefficients directly depend on the intensity rather than the gradient of the edge graph, thereby greatly reducing the computational complexity.

**Figure 1:**
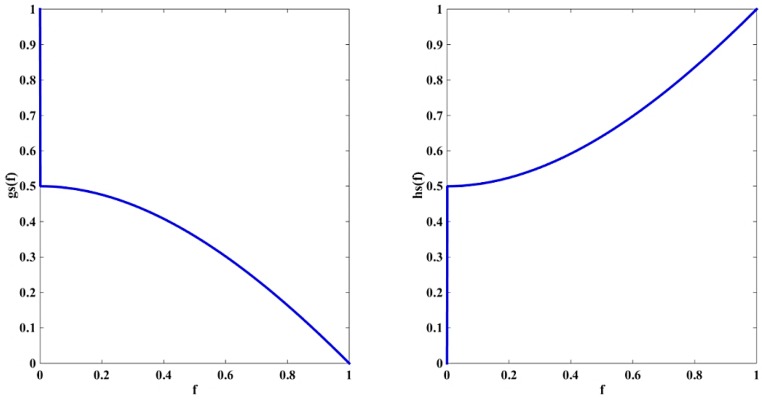
Variation of coefficients when τ = 1.

Moreover, as shown in Figure 1, the variation of the parameters in Equations (15) and (16) with the intensity of the edge graph takes the form of convex function. Compared with the parameters α and β in NBGVF (Equations (10) and (11)), the coefficients of the proposed model change gradually when the value of f is high, and thus offer more protection to the weak edge in the infrared images. Hence, the proposed model is capable of segmenting the infrared images more accurately. Meanwhile, the coefficients of the proposed model fluctuate violently when the value of f is low. As a result, contour divergence is more efficient at a long distance from the edge.

**Figure 2:**
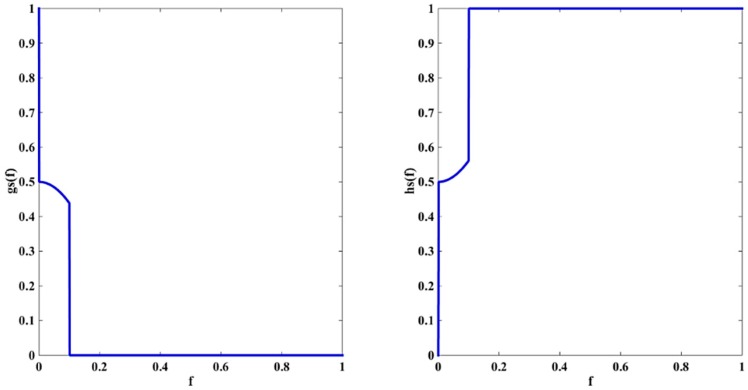
Variation of coefficients when τ = 0.1.

After the parameter in the equation is set to 0.1, the variation of the two coefficients is shown in Figure 2. This figure shows that the two coefficients fluctuate violently, dwindle to zero and then jump to 1 when the intensity increases. This means that the diffusion of the image near the edge graph along the normal direction is inhibited quickly, and that only the tangent diffusion component is left finally. This type of variation is conducive to the protection of weak edges. As discussed above, the value of K should increases with the image noise to smooth the noise, but the weak edge is likely to be lost due to noise smoothing. Hence, the value of the parameter should be optimized to achieve a trade-off between noise smoothing and weak edge protection.

### 3.2. Numerical Implementation

Now, the external force field can be obtained by minimizing Equation (12). The Euler-Lagrange equation of the energy functional can be written as:

(17) $$g\cdot \left(gs\cdot V_{NN}+hs\cdot V_{TT}\right)+\;h\cdot \left(V-\nabla f\right)=0$$

In order to obtain the vector field in Equation (17), we introduce the parameter t and construct the following partial differential equation.

(18) $$\frac{\partial V}{\partial t}=g\cdot \left(gs\cdot V_{NN}+hs\cdot V_{TT}\right)+\;h\cdot \left(V-\nabla f\right)$$

(19) $$\{\begin{array}{c} V_{NN}=\frac{1}{{\left|\nabla V\right|}^{2}}\left(V_{x}^{2}V_{yy}+V_{y}^{2}V_{xx}-2V_{x}V_{y}V_{xy}\right)\\ V_{TT}=\frac{1}{{\left|\nabla V\right|}^{2}}\left(V_{x}^{2}V_{yy}+V_{y}^{2}V_{xx}+2V_{x}V_{y}V_{xy}\right) \end{array}$$

where $V_{x}V_{y}$ is the first-order partial derivative with respect to x or y, $V_{xx}V_{yy}$ is the second-order partial derivative with respect to x or y, and $V_{xy}$ is the result achieved by computing the partial derivative with respect to x and then to y.

The equations above can be solved by finding the equilibrium solution to the following set of partial differential equations:

(20) $$\{\begin{array}{c} u_{t}=g\cdot \left(gs\cdot u_{NN}+hs\cdot u_{TT}\right)+\;h\cdot \left(u-f_{x}\right)\\ v_{t}=g\cdot \left(gs\cdot v_{NN}+hs\cdot v_{TT}\right)+\;h\cdot \left(v-f_{y}\right) \end{array}$$

where $u=\frac{\partial V}{\partial x}$, $v=\frac{\partial V}{\partial y}$, $f_{x}=\frac{\partial f}{\partial x}$, and $f_{y}=\frac{\partial f}{\partial y}$. Iterating Equation (20) yields the desired external force field. Hence, the evolution equation of this external force field can be written as:

(21) $$V_{t}\left(x,y,t\right)=g\left(\left|\nabla f\right|\right)\left(gs\left(f\right)V_{NN}\left(x,y,t\right)+hs\left(f\right)V_{TT}\left(x,y,t\right)\right)-h\left(\left|\nabla f\right|\right)\left[V\left(x,y,t\right)-\nabla f\right]$$

**Figure 3:**
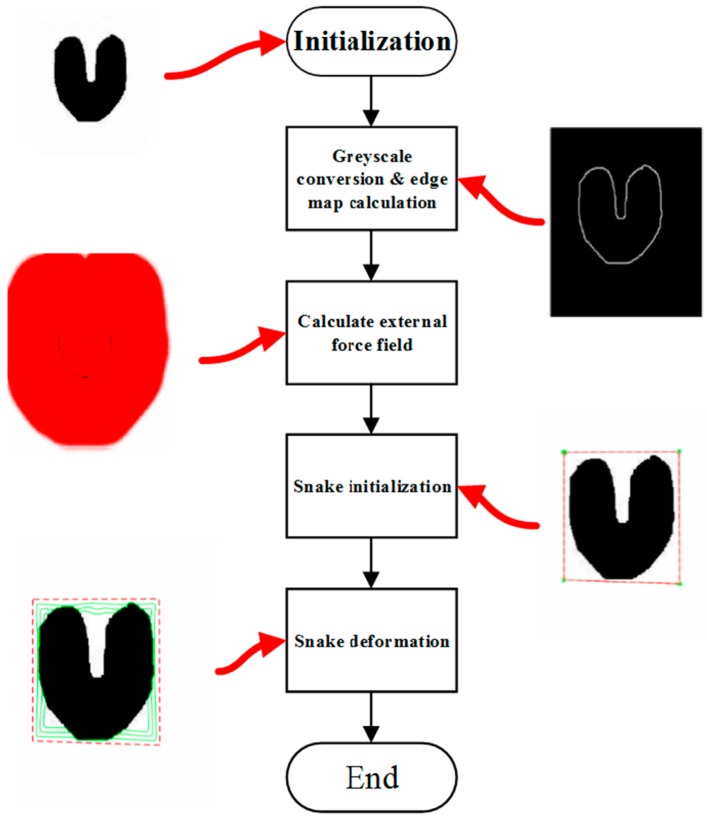
The flowchart of the algorithm.

The algorithm steps are given in Figure 3.

## 4. Experimental Results and Analysis

In this section, the proposed GVF model will be compared with GVF [18], GGVF [19], NGVF [20], NBGVF [21], CN-GGVF [6], LIF [28] and SOAC [29] across different images. First, we apply these methods to standard images, including the U-shaped image and the LTI image. All of these images are the traditional images used to evaluate the basic performance of various Snakes models. Afterwards, we will evaluate the performance of the proposed model and other algorithms in terms of segmenting infrared images, such as the original infrared image and the infrared images corrupted with various types of noises. These segmentation results form the basis for detailed analysis and comparison. MATLAB R2014B is used as the development environment of the experiment programs in this paper. The computer configuration is Inter Core i5-4210M 2.6 GHz CPU and 8 GB RAM.

Subjective assessment has its limitations for the evaluation of segmentation performance. In our experiments, the segmentation results are evaluated using the following metrics: Precision, Recall and F1 measure [1]. Let $M_{seg}$ denote the actual segmentation results and $G_{seg}$ denote the segmentation baseline.

The metric Prevision can be expressed as:

(22) $$P=\frac{M_{seg}\cap G_{seg}}{M_{seg}}$$

Similarly, Recall can be defined as:

(23) $$R=\frac{M_{seg}\cap G_{seg}}{G_{seg}}$$

F1 measure provides an evaluation metric that combines Precision with Recall. It is defined as:

(24) $$F=\frac{2\times P\times R}{P+R}$$

A high value for any of these three metrics means that the segmentation is accurate and the result approximates to the ground truth.

### 4.1. Catching Range, Convergence for Convex and Concave Planes and Insensitivity to Initial Contours

**Figure 4:**
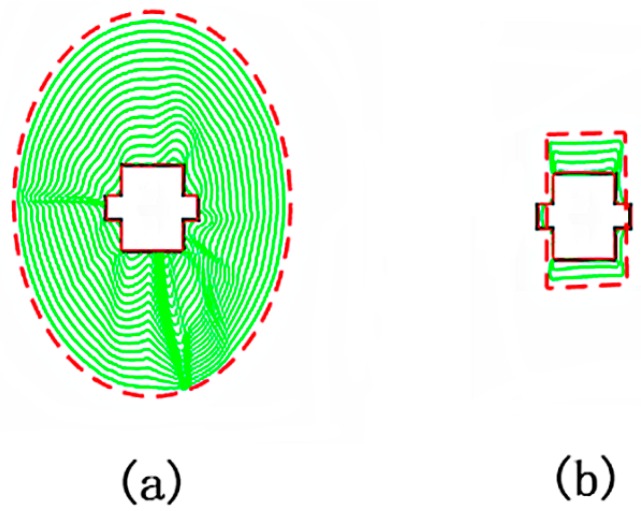
(**a**) Evolution of the contour when the initial contour is large; and (**b**) evolution of the contour when the initial contour is small.

**Figure 5:**
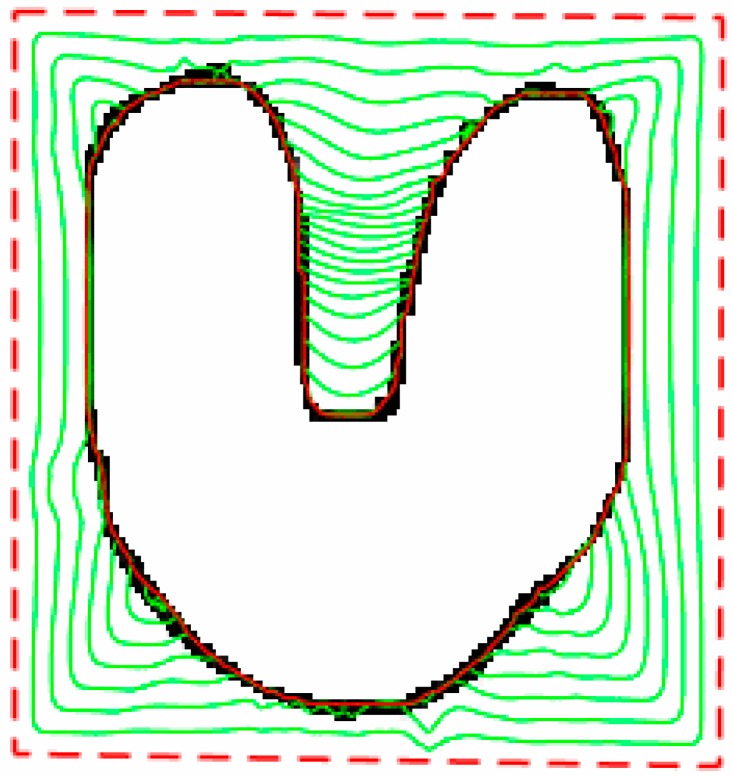
Segmentation results of U-shape image.

In this set of experiments, we use the U-shaped and square images to test the performance of the proposed method. The contour of the proposed method evolves from a long distance away towards the target edge of the image. The parameter setting of the proposed method is {K,τ}={0.1, 1} and the evolution is shown in Figure 4 and Figure 5. It can be seen that the final contour is well matched with the target edge. The results in Figure 4a show the large catching range of the proposed model. The results in Figure 4a,b demonstrate the ability of the proposed method to obtain accurate segmentation results regardless of where the initial contour is placed and whether the contour is distant from the object or passes through the target edge.

Figure 5 demonstrates the ability of the proposed method to converge for convex and concave planes and obtain the U-shaped edge accurately through segmentation.

### 4.2. Convergence for Long Narrow Edges

**Figure 6:**
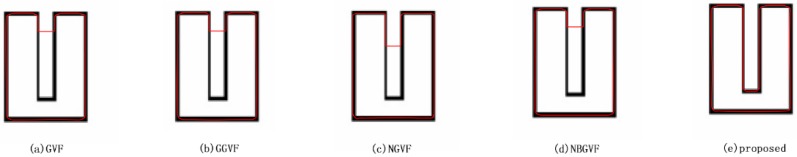
LTI (Long and Thin Indentation) convergence results of all models.

In this subsection, the proposed method will be evaluated and compared with other traditional active contour models in terms of LTI image segmentation. The parameter setting of the proposed method is {K,τ}={1, 0.5}. The classic LTI images can test the convergence performance of the active contour model in the case of long narrow edges. The experimental results are given in Figure 6. These results clearly show that only the proposed method has the ability to converge towards the bottom of the LTI images, while the other algorithms stop converging at the entrance to the LTI images. Hence, the proposed algorithm has remarkable superiority over the traditional models.

### 4.3. Parameter Settings Sensitivity

**Figure 7:**
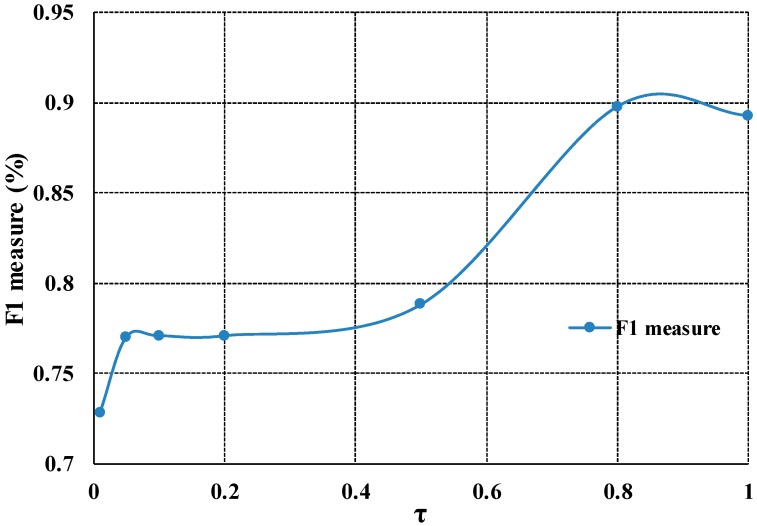
The observation of relationship between segmentation accuracy and the value of τ (the valve of K is constant “1”).

To demonstrate the parameter sensitivity of our proposed model, we change the parameter “τ” from 0.01 to 1 and obtain the experiment results. Next, the results are quantitative calculated by the F1 measure criterion. Then we obtain the average F1 measure value of the experiment. Finally, the following curve in Figure 7 is acquired. According to Figure 7, in the range of 0.1 to 0.5 and 0.8 to 1, the F1 measure values change is not so obvious. Thus, our proposed model can be insensitive to parameter settings in the certain range.

### 4.4. Segmentation Results for Common Real-World Infrared Images

In this subsection, we will use the infrared images to evaluate the comprehensive performance of the proposed model. We captured the infrared images of the airplane, ship and tank using the infrared camera at a resolution of 640 × 480. After being pre-processed, including grayscale conversion and edge map calculation, the images are segmented using the proposed model and other traditional algorithms. The segmentation results are then compared.

**Figure 8:**
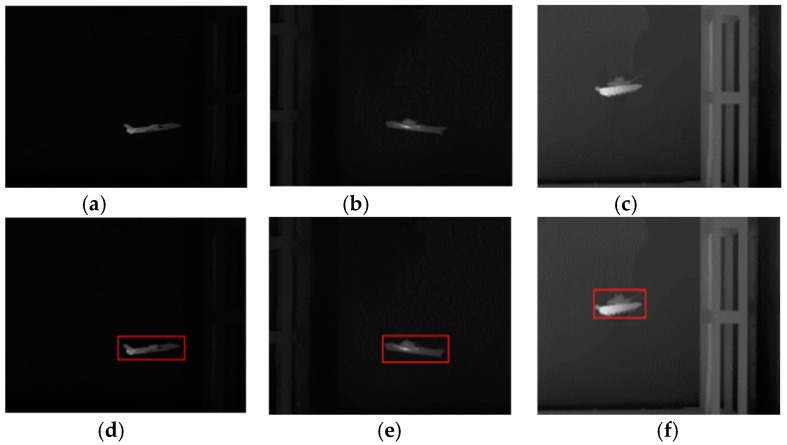
Original images and initial contours used in the experiment. (**a**) “plane”; (**b**) “ship”; (**c**) “tank”; (**d**) Initial contour of “plane” (size: 165 × 75); (**e**) Initial contour of “ship” (size: 158 × 86); (**f**) Initial contour of “tank” (size: 123 × 98).

**Figure 9:**
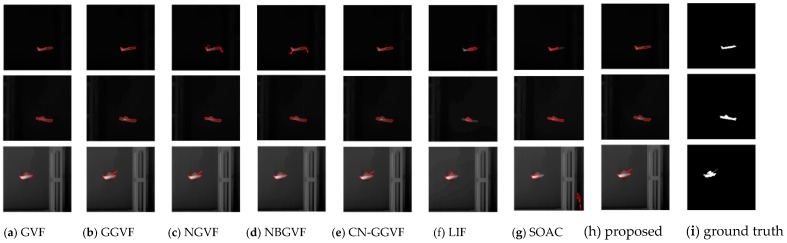
Segmentation results of usual infrared images.

In the experiment, the parameter setting of the proposed model is {K,τ}={0.2, 1}. The major influences that affect segmentation accuracy are the weak target edges and the interference from the edges of other objects near the target. Figure 8 shows the original infrared images used in the experiment and Figure 9 shows the segmentation results of various active contour models. The last column is the ground truth. From Figure 9, it can be seen intuitively that the propose model can segment the infrared images very accurately and is superior to other traditional models in terms of accuracy. As discussed at the beginning of this section, subjective evaluation has some limitations. Hence, we perform quantitative analysis of these results based on Equations (21)–(23).

The data in Table 1 intuitively reveal the advantages and disadvantages of the proposed method over other algorithms in terms of infrared image segmentation. Consider the metric of F1 measure, which can reflect the segmentation performance overall. The value of this metric of the proposed method is higher than the other algorithms across the three images. This demonstrates the undisputed superiority of the proposed method.

### 4.5. Segmentation Results of Noise-Corrupted Infrared Images

**Figure 10:**
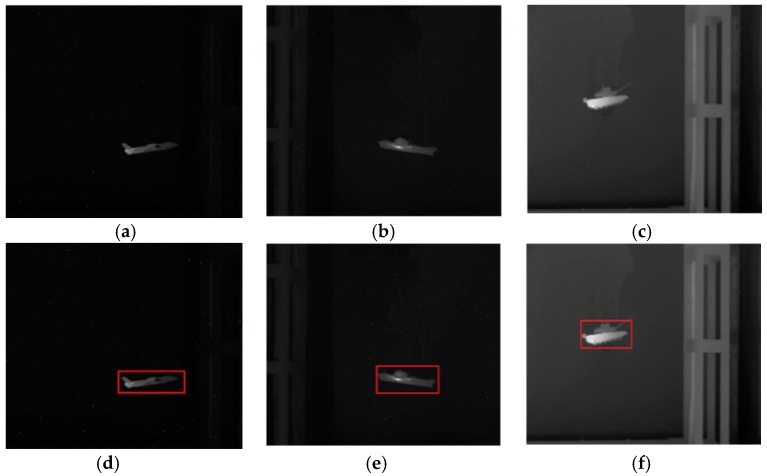
Images corrupted with the salt–pepper noises and initial contours in the experiment. (**a**) “planeN”; (**b**) “shipN”; (**c**) “tankN”; (**d**) Initial contour of “planeN” (size: 160 × 72); (**e**) Initial contour of “shipN” (size: 155 × 85); (**f**) Initial contour of “tankN” (size: 120 × 95).

**Figure 11:**
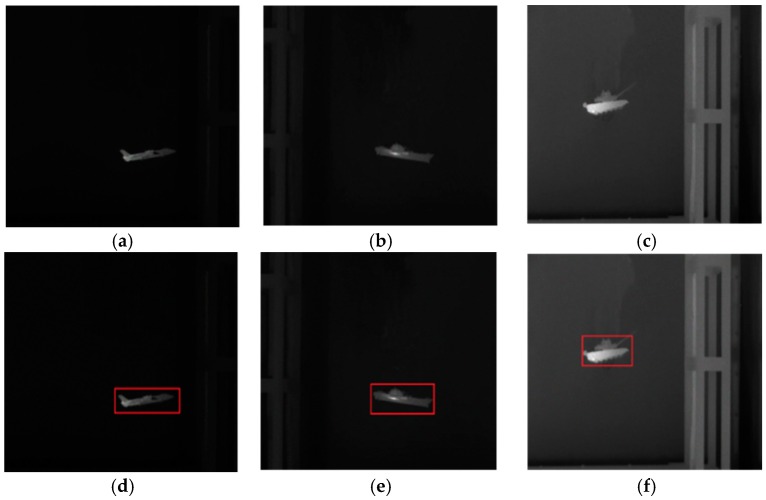
Images corrupted with the multiplicative noises and initial contours in the experiment. (**a**) “planeN2”; (**b**) “shipN2”; (**c**) “tankN2”; (**d**) Initial contour of “planeN2” (size: 170 × 75); (**e**) Initial contour of “shipN2” (size: 156 × 85); (**f**) Initial contour of “tankN2” (size: 125 × 95).

The images used in the experiment of the previous subsection were captured in the experimental environment and were processed specifically. However, in the real-world applications, the images we obtain are mostly corrupted with the noise, thereby expecting the proposed algorithm to smooth these noises. In order to verify the proposed algorithm’s insensitivity to the noise, we corrupt the original infrared images with the noise using the “imnoise” function in MATLAB. In this subsection, we will use these noise-corrupted images for experiments. We add the salt–pepper and multiplicative noises to the original images. The images corrupted with the salt–pepper noise are named planeN, shipN and tankN (Figure 10), and the parameter setting of “D” (noise density) is 0.001. The images corrupted with the multiplicative noise are named planeN2, shipN2 and tankN2 (Figure 11), and the parameter setting of “V” (variance) is 0.01.

**Figure 12:**
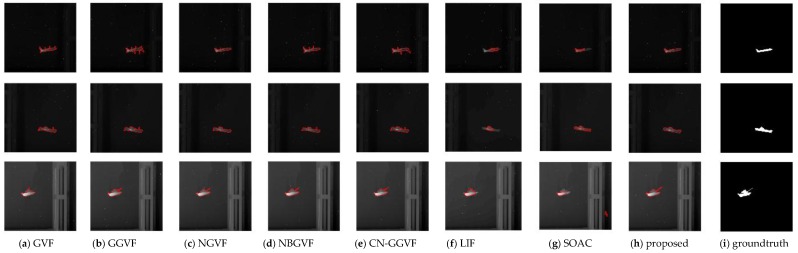
Segmentation results of the images corrupted with salt–pepper noises.

**Figure 13:**
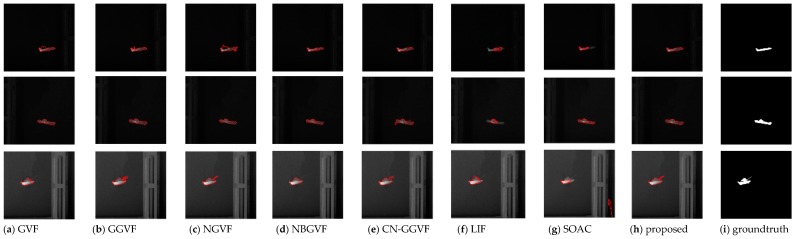
Segmentation results of the images corrupted with multiplicative noises.

The parameter setting of the proposed model is  {K,τ}={0.1, 0.2}. In addition to the two major influences discussed in previous subsection, the noise is another factor that affects segmentation accuracy. For some models such as GVF and NBGVF, there is a trade-off between noise smoothing and weak edge protection. This is particularly true for images ship and tank. Some models tradeoff weak edge protection for noise smoothing for the purpose of obtaining better results. However, the proposed model is capable of protecting weak edges without sacrifice of noise smoothing, thereby resulting in greater accuracy. Figure 12 and Figure 13 shows the segmentation results of various active contour models. As in the previous subsection, we perform quantitative analysis of experimental results using the same three metrics (Precision, Recall, and F1 measure). The analysis results are given in the Table 2 and Table 3.

The data show that after the noise is added to the image, the proposed algorithm can still segment images very accurately, as the value of each metric is above 0.9. The accuracy of the proposed method is very close or even higher than the best algorithm, and the proposed method leads the worse algorithms by a larger margin. These results demonstrate the ability of the proposed method to smooth noises in the infrared images more effectively.

To prove our proposed model can be applied to infrared images with different noise intensity, we add different multiplicative noise to the infrared image “plane” by changing the parameter “V” in the “imnoise” function from 0.01 to 0.03 (with the gap of 0.005), and then apply our proposed model to these noise-polluted images and obtain the test results.

**Figure 14:**
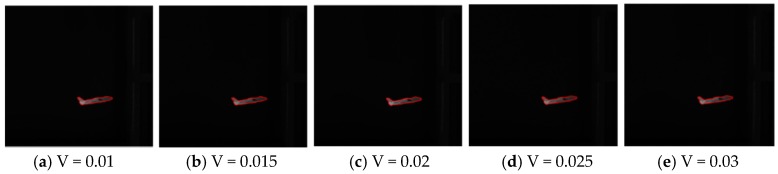
Segmentation results in the infrared images with different ‘V’ values of noise intensity. (The noise intensity gets higher from left to right.)

According to Figure 14, we can clearly see that the segmentation results are almost the same in the different images. Thus, the results prove that our proposed model can process different noise intensity adaptively. Our proposed model is very robust to the noise.

### 4.6. Discussion

**Figure 15:**
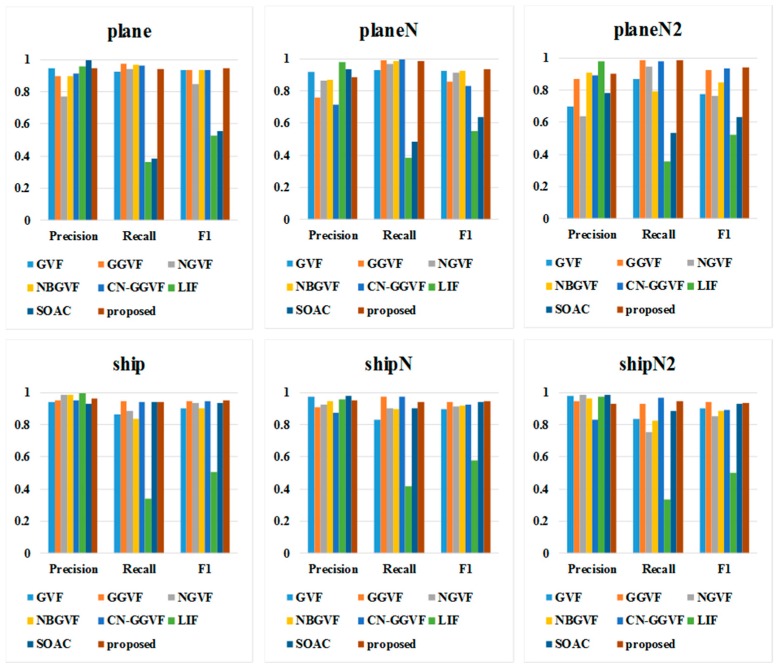

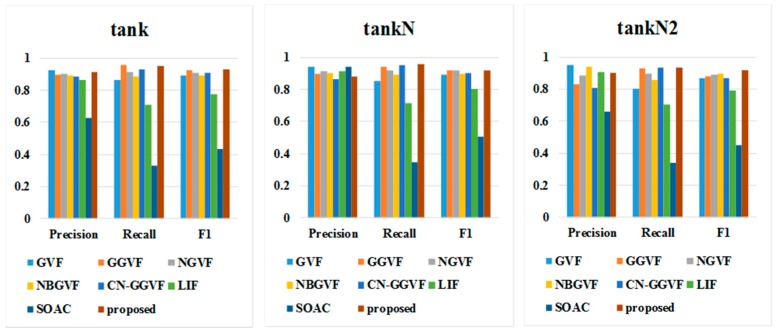
Block diagrams of quantitatively analyzed segmentation results of infrared images.

The block diagrams in Figure 15 clearly show the segmentation accuracy of the proposed method and other algorithms. It can be seen that the proposed method is superior to other algorithms for most of the infrared images and is almost insensitive to the noise. The average CPU time and number of iterations in these experiments are shown in Table 4.

**Figure 16:**
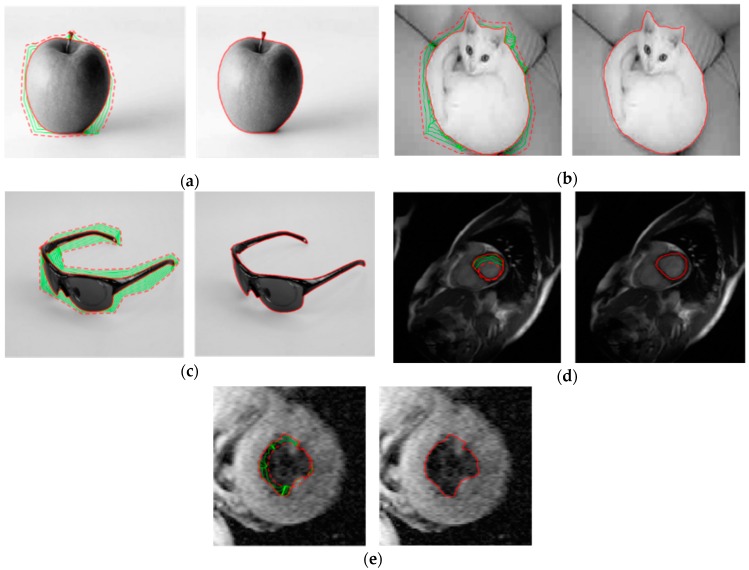
Evolution of the contour and segmentation results of natural images and medical images using proposed method.

In order to prove that the proposed method is suitable for more applications, we apply the proposed method to natural images in several sets of experiments. The results are shown in Figure 16.

We can see that the proposed method is still capable of segmenting the natural images satisfactorily. Hence, the high segmentation accuracy qualifies the proposed method for more applications.

To sum up, we first perform experiments to verify some basic properties of the proposed method and prove that the proposed method is vastly superior to other classic algorithms in terms of LTI convergence. Afterwards, we apply the proposed method to the infrared images and compare with classic algorithms. Results show that the proposed method has great advantages over other algorithms. Finally, we evaluate the segmentation performance of the proposed method for natural images. Results imply that the proposed method is suitable for more applications.

## 5. Conclusions

The infrared image segmentation technology is of great significance to real-world life and manufacturing. However, many issues have yet to be addressed. The research on the use of active contour model for infrared image segmentation is in the infancy, but it has attracted a lot of attention. In this paper, we adapt the active contour model to the infrared images by improving NBGVF. A series of experiments have been performed to prove segmentation accuracy superiority of the proposed method over other algorithms (GVF, GGVF, NGVF, NBGVF, CN-GGVF, LIF, and SOAC). Meanwhile, it is proven that the proposed method can smooth noises while protecting weak edges in the infrared images. Hence, the proposed method is vastly superior to other algorithms.

## Figures and Tables

**Table 1:** Quantitative analysis results of usual infrared images after segmentation.

		GVF	GGVF	NGVF	NBGVF	CN-GGVF	LIF	SOAC	Proposed
plane	Precision	0.9475	0.8958	0.7723	0.8984	0.914	0.955	0.9948	0.9484
	Recall	0.9246	0.9759	0.9407	0.9688	0.9618	0.3628	0.3829	0.9427
	F1	0.9359	0.9341	0.8482	0.9323	0.9373	0.5259	0.553	0.9456
ship	Precision	0.9398	0.9497	0.9859	0.9835	0.9494	0.9961	0.9269	0.9597
	Recall	0.8645	0.9453	0.8858	0.8331	0.9399	0.3391	0.9399	0.9386
	F1	0.9006	0.9475	0.9332	0.9021	0.9446	0.506	0.9334	0.949
tank	Precision	0.925	0.8972	0.8993	0.8929	0.8846	0.8625	0.6616	0.9122
	Recall	0.8621	0.9549	0.9116	0.8868	0.9295	0.7073	0.3389	0.9505
	F1	0.8924	0.9251	0.9054	0.8899	0.9065	0.7772	0.4482	0.931

**Table 2:** The comparison of Quantitative evaluation results on infrared images corrupted with salt–pepper noise.

		GVF	GGVF	NGVF	NBGVF	CN-GGVF	SOAC	LIF	Proposed
planeN	Precision	0.9177	0.7606	0.8643	0.8705	0.7147	0.9337	0.9769	0.8861
	Recall	0.9296	0.9899	0.9668	0.9859	0.996	0.4814	0.3819	0.9849
	F1	0.9236	0.8603	0.9127	0.9246	0.8322	0.6353	0.5491	0.9329
shipN	Precision	0.9756	0.9097	0.9262	0.944	0.8767	0.9798	0.9555	0.9527
	Recall	0.8284	0.9753	0.9045	0.8963	0.9733	0.9045	0.4159	0.9406
	F1	0.896	0.9414	0.9152	0.9195	0.9225	0.9406	0.5795	0.9466
tankN	Precision	0.9381	0.8944	0.9145	0.9027	0.8607	0.9407	0.9104	0.8815
	Recall	0.8528	0.9431	0.9196	0.8893	0.9518	0.3432	0.7168	0.9567
	F1	0.8934	0.9181	0.9171	0.896	0.904	0.5029	0.8021	0.9176

**Table 3:** The comparison of Quantitative evaluation results on infrared images corrupted with multiplicative noise.

		GVF	GGVF	NGVF	NBGVF	CN-GGVF	SOAC	LIF	Proposed
planeN2	Precision	0.6992	0.8713	0.6366	0.9076	0.8928	0.7794	0.978	0.9026
	Recall	0.8714	0.9869	0.9477	0.793	0.9789	0.5327	0.3568	0.9869
	F1	0.7758	0.9255	0.7616	0.8464	0.9338	0.6328	0.5228	0.9429
shipN2	Precision	0.9805	0.9455	0.987	0.9598	0.8277	0.9852	0.9711	0.9277
	Recall	0.8336	0.9317	0.7533	0.8223	0.9662	0.884	0.3342	0.9443
	F1	0.9011	0.9385	0.8545	0.8857	0.8887	0.9318	0.4973	0.9359
tankN2	Precision	0.9487	0.8297	0.8835	0.9383	0.8086	0.6616	0.9082	0.9037
	Recall	0.8002	0.9307	0.8955	0.8565	0.9326	0.3389	0.7038	0.9338
	F1	0.8682	0.8773	0.8894	0.8956	0.8662	0.4482	0.793	0.9185

**Table 4:** Average CPU time and number of iterations in the experiments of Section 4.4 and Section 4.5.

	GVF	GGVF	NGVF	NBGVF	CN-GGVF	LIF	SOAC	Proposed
**Average CPU Time (s)**	81.131	84.074	93.401	88.699	86.297	184.065	117.122	79.561
**Number of Iterations**	100	100	100	100	100	300	200	100
